# Supplementary material for: Dose Intervals and Time since Final Dose on Changes in Metabolic Indices after COVID-19 Vaccination
Source: Vaccines (Basel). 2023 Nov 23;11(12):1746. doi: 10.3390/vaccines11121746 (PMC10748310; doi:10.3390/vaccines11121746)
Supplement: Supplementary file 1 [file vaccines-11-01746-s001.zip › vaccines-2686407-supplementary.pdf]

Table S1: Mean and Median differences (post-vaccination – pre-vaccination) among groups based on the first-to-second-dose dose interval and time since the final dose along with p-values.

	Changes in 1st to 2nd dose interval				Changes in the time since the final dose			
	≤ 8 Weeks	*p*	> 8 Weeks	*p*	≤ 6 months	*p*	> 6 months	*p*
**Adolescents**								
Weight (Kg)	11.5	<0.001	6.99	0.004	9.1	0.006	10.67	<0.001
BMI (Kg/m^2)	4.83	<0.001	3.15	0.002	3.78	0.007	4.57	<0.001
Waist (cm)	6.24	<0.001	4.75	0.106	7.6	0.001	5.16	0.001
Hips (cm)	8.57	<0.001	7.09	0.006	6.11	0.009	8.92	<0.001
Systolic BP (mmHG)	5.18	0.002	0.78	0.981	-3.5	0.349	6.74	<0.001
Diastolic BP (mm HG)	3.1	0.018	3.03	0.048	-2.64	0.337	5.26	<0.001
Total Chol (mmol/l)	0.09	0.294	-0.08	0.539	0.12	0.018	-0.06	0.491
Fasting glucose (mmol/l)	-0.02	0.729	-0.04	0.818	-0.13	0.462	0	0.927
HbA1c	-0.07	0.388	-0.11	0.494	-0.24	0.138	-0.03	0.763
HDL-Chol (mmol/l)	0.27	<0.001	0.13	0.069	0.29	<0.001	0.21	<0.001
Triglycerides (mmol/l)	0.75	<0.001	0.67	<0.001	0.57	0.001	0.75	<0.001
25(OH)D (nmol/l)	7.2	<0.001	10.1	0.004	10.05	<0.001	7.47	<0.001
**Adults**								
Weight (Kg)	2.46	0.023	0.96	0.251	1.7	0.022	1.22	0.337
BMI (Kg/m^2)	0.97	0.017	0.4	0.216	0.66	0.021	0.51	0.281
Waist (cm)	-3.17	0.076	-3.94	0.002	-3.57	0.003	-3.78	0.049
Hips (cm)	-4.81	0.013	-8.28	<0.001	-7.42	<0.001	-6.18	0.004
Systolic BP (mmHG)	-9.76	<0.001	-8.03	<0.001	-7.53	<0.001	-10.67	<0.001
Diastolic BP (mm HG)	-2.64	0.085	-2.76	0.031	-3.4	0.007	-1.53	0.325
Total Chol (mmol/l)	0.33	0.016	0.28	0.003	0.36	<0.001	0.22	0.097
Fasting glucose (mmol/l)	-0.13	0.543	-0.07	0.727	-0.21	0.254	0.12	0.616

HbA1c	0.2	0.113	0.36	0.002	0.35	0.002	0.21	0.098
HDL-Chol (mmol/l)	0.38	<0.001	0.42	<0.001	0.45	<0.001	0.35	<0.001
Triglycerides (mmol/l)	-0.1	0.609	-0.01	0.479	-0.11	0.009	0.14	0.098
25(OH)D D(nmol/l)	-4.27	0.597	12.48	0.056	6.75	0.271	0.35	0.114

Note: The data are presented as mean and median differences (post-vaccination – pre-vaccination) in the groups for continuous normal and continuous non-normal variables, along with the associated p-values. P<.05 was considered statistically significant.

Table S2: Pre- to post-vaccination changes in the prevalence of MetS and its components by study groups and the associated p-values.

**Mets components**	**Pre- Vaccination**	**Post- Vaccination**	**1st to 2nd dose (weeks)**				**Time to final dose (months)**			
			≤ 8 weeks	*p*	> 8 weeks	*p*	≤ 6 months	*p*	> 6 months	*p*
**Adolescents**										
Central Obesity	7 (5.3)	24 (18.2)	13.6	0.002	10.9	0.219	13.8	0.125	12.5	0.004
Hyperglycemia	12 (9.1)	17 (12.9)	0	1	13.5	0.125	0	1	5.2	0.302
Low HDL	82 (62.1)	53 (40.2)	-25.3	0.001	-13.6	0.332	-20.6	0.003	-14.5	0.054
High Triglycerides	26 (19.7)	85 (64.4)	47.4	<0.001	37.9	0.001	30.6	0.007	50	<0.001
Hypertension	30 (22.7)	28 (21.2)	-5.3	0.383	8.1	0.549	-16.7	0.109	4.2	0.523
Mets	11 (8.3)	22 (16.7)	4.2	0.481	18.9	0.039	0	1	11.4	0.013
MetS comp.	1.19 ± 1.0	1.57 ± 1.0	0.31	0.032	0.57	0.014	-0.14	0.436	0.58	<0.001
**Adults**										
Central Obesity	46 (24.7)	39 (21.0)	1.4	0.991	-6.9	0.134	-5.9	0.143	0	1
Hyperglycemia	47 (25.3)	35 (18.8)	-7.2	0.227	-6	0.296	-10.2	0.036	0	1
Low HDL	147 (79.0)	55 (29.6)	-47.1	<0.001	-50.8	<0.001	-55.1	<0.001	-39.7	<0.001
High Triglycerides	48 (25.8)	41 (22.0)	-4.3	0.629	-3.5	0.636	-11.9	0.002	10.3	0.621
Hypertension	54 (29.0)	14 (7.5)	-21.4	0.003	-21.5	<0.001	-22.9	<0.001	-19.2	0.007
MetS	48 (25.8)	21 (11.3)	-12.8	0.022	-15.5	0.005	-16.1	0.001	-11.8	0.115
MetS comp.	1.84 ± 1.2	0.99 ± 1.1	-0.79	<0.001	-0.89	<0.001	-1.06	<0.001	-0.49	0.017

Note: The prevalence of MetS and its components is presented by N (%). The pre-and post-vaccination changes are presented by the prevalence percentage from pre-vaccination to post-vaccination visits, calculated for all four study groups. P<0.05 was considered statistically significant.

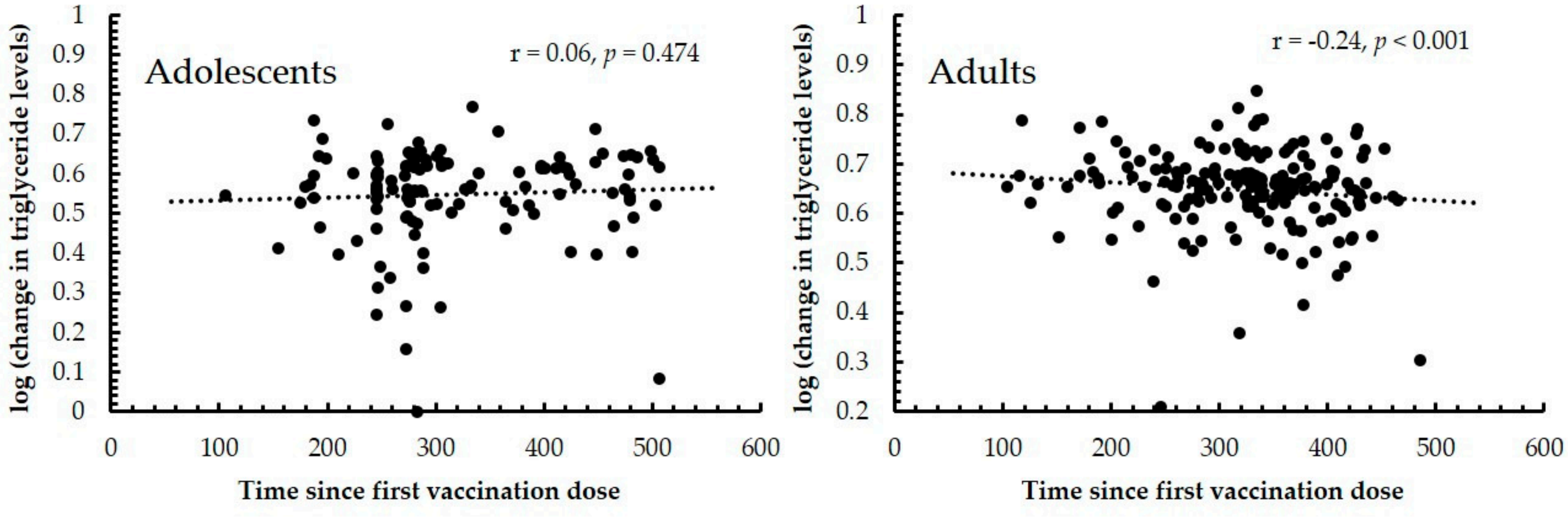

Figure S1. Scatterplot depicting the correlation between time since first dose and pre to post vaccination changes in triglyceride levels.
